# Genetic polymorphism of the human organic solute carrier protein 1 (hOSCP1) gene in Japanese patients with non-viral liver carcinoma

**DOI:** 10.1016/j.mgene.2014.09.002

**Published:** 2014-10-01

**Authors:** Mayumi Toda, Yasuna Kobayashi, Tomotake Koizumi, Koji Saito, Masayuki Ohbayashi, Noriko Kohyama, Takeshi Aoki, Masahiko Murakami, Hajime Yasuhara, Toshinori Yamamoto

**Affiliations:** a Department of Pharmacotherapeutics, Division of Clinical Pharmacy, Showa University, 1-5-8 Hatanodai, Shinagawa-ku, Tokyo 142-8555, Japan; b School of Pharmacy, 2nd Department of Pharmacology, Showa University, 1-5-8 Hatanodai, Shinagawa-ku, Tokyo 142-8555, Japan; c School of Medicine, Department of Pathology, Division of Pathology, Showa University, 1-5-8 Hatanodai, Shinagawa-ku, Tokyo 142-8555, Japan; d School of Medicine, Department of Gastroenterological and General Surgery, Showa University, 1-5-8 Hatanodai, Shinagawa-ku, Tokyo 142-8555, Japan

**Keywords:** AGC2, aspartate glutamate carrier 2, ALT, alanine aminotransferase, AST, aspartate aminotransferase, cSNPs, coding single nucleotide polymorphisms, DNA, deoxyribonucleic acid, γ-GTP, γ-glutamyltranspeptidase, HCC, hepatocellular carcinoma, HCV, hepatitis C virus, hOSCP1, human organic solute carrier protein 1, hURAT1, urate transporter 1, HWE, Hardy–Weinberg equilibrium, ICC, intrahepatic cholangiocarcinoma, ICG, indocyanine green test, LC, liver carcinoma, LDH, lactate dehydrogenase, MDR1, multidrug-resistance 1, NAFLD, non-alcoholic fatty liver disease, OAT, organic anion transporter, OATP, organic anion transporting polypeptide, PCR, polymerase chain reaction, SLC/Slc, solute carrier, SNPs, single nucleotide polymorphisms, hOSCP1, Non-viral liver carcinoma, Genetic polymorphism, Transporter

## Abstract

Human organic solute carrier protein 1 (hOSCP1) is a Na^+^-independent multispecific organic solute transporter. To date, several studies have revealed that gene mutations of the transporters are likely to be associated with some diseases; however, there are no data concerning the genetic polymorphism of the *hOSCP1* gene in Japanese patients with non-viral liver carcinoma (LC). In the present study, we isolated genomic DNA from a normal portion of LC, and analyzed 41 single nucleotide polymorphisms (SNPs) chosen from a database of SNPs (dbSNPs). We found genotype frequencies for 2 non-synonymous SNPs [rs34409118 (Thr^131^ → Ala) and rs1416840 (Ile^219^ → Thr)] and 1 synonymous SNP [rs16822954 (Ser^193^ → Ser)] to be statistically significant when compared with dbSNPs. No statistical significance was observed in rs2275477 (Gly^307^ → Arg) in the *hOSCP1* gene. With respect to the allele frequency, we also observed rs34409118 to be statistically significant. Interestingly, we found that non-viral LC patients do not carry heterozygous mutations in rs1416840 (A/G) and rs16822954 (A/G), suggesting that a non-carrier of heterozygous mutations in these two SNPs might be a biomarker for susceptibility for non-viral LC in Japanese. Further analyses of patients with hOSCP1 variants may elucidate the relationship between the *hOSCP1* gene and susceptibility of non-viral LC in Japanese patients.

## Introduction

Liver cancer (LC), one of the most common and deadly cancers, as well as advanced colon and gastric cancers, is a major cause of cancer death (Parkin et al., 2005). Most LC invariably arises in the setting of chronic progressive liver diseases specifically chronic infections by hepatitis C viruses (HCV) (Colombo, 1992, Okuda, 1992). HCV infection is believed to be a major cause of LC (De Bac et al., 1994) and is associated with > 70% of LC in Japan (Tanaka et al., 1991). In about 20% of patients, chronic hepatitis C progresses to liver cirrhosis, which is complicated by hepatocellular carcinoma (HCC) development (Kiyosawa et al., 1990). Although HCV infection is strongly correlated with LC development (Bartosch et al., 2009), non-viral factors also play important roles in enhancing liver cancer progression. To date, several risks such as non-alcoholic fatty liver disease (NAFLD) (Caldwell and Crespo, 2004), obesity (Blonski et al., 2010, Wolk et al., 2001), diabetes (El-Serag et al., 2004), alcohol consumption (Donato et al., 2002), diet (Polesel et al., 2007) and cigarette smoke history (Marrero et al., 2005) are important factors for the development of non-viral LC. In addition, hepatic cancer risk can be influenced by exposure to small molecules of xenobiotics such as aflatoxin (Qian et al., 1994) and pesticides (Anwar et al., 2008). However, these factors do not fully account for the observed clinical variability of non-viral LC occurrence.

The plasma membrane of epithelial cells plays a pivotal role in the transport of a wide range of organic solutes including drugs and chemicals (VanWert et al., 2010). In 2005, we succeeded in isolating a novel gene encoding human organic solute carrier 1 (hOSCP1) (also known as oxidored-nitro domain-containing protein 1, NOR1) (Kobayashi et al., 2005). hOSCP1 cDNA consists of 1137 base pairs that encode 379 amino acid sequences and mediate the high affinity transport of both anionic and cationic compounds. Subsequently, we have isolated mouse and rat homologs of Oscp1 (Izuno et al., 2007, Kobayashi et al., 2007). With respect to the genetic polymorphism of the *hOSCP1* gene, Nie et al. reported that non-synonymous SNP (Glu^58^ → Gly) of the NOR1 may be involved in the development and/or progression of nasopharyngeal carcinoma (Nie et al., 2003). Although the expression of hOSCP1 mRNA exhibited quite low level in the human liver (Kobayashi et al., 2005), we assumed that genetic variation of the gene coding for hOSCP1 might be related to the development and/or progression of other carcinomas such as non-viral LC as observed by Nie et al. (2003). In addition, there are no data concerning whether genetic variation of the *hOSCP1* gene impacts the risk of non-viral LC.

In the present study, therefore, we examined whether genetic variants in the *hOSCP1* gene are associated with the incidence of non-viral LC in Japanese.

## Materials and methods

### Patients

Normal portions of human liver (noncancerous liver tissues) were obtained from 18 Japanese hepatectomized patients with non-viral LC at Showa University Hospital. The liver samples were numbered anonymously. All patients gave their written informed consent. This study was conducted in accordance with the Declaration of Helsinki and its amendments and was approved by the Human Genome Ethics Committee, Showa University.

### Genomic DNA extraction and genotyping of hOSCP1

Genotyping was investigated by direct sequencing method. After being classified histopathologically, normal portions of the livers were snap-frozen in liquid nitrogen and stored at − 80 °C for subsequent genomic DNA extraction. Genomic DNA was extracted from resected normal portions of the liver tissues using a Genomic DNA from Tissue kit (Macherey-Nagel Inc., Bethlehem, PA, U.S.A.). Ten coding SNPs (cSNPs) in the *hOSCP1* (accession# AB079075) gene were chosen from a database of dbSNP (http://www.ncbi.nlm.nih.gov/snp/). We also analyzed neighbor SNPs of those selected cSNPs (± 100 base pair).

### PCR and sequence analysis

Oligonucleotides for polymerase chain reaction (PCR) were designed using a Primer3 Output program (http://bioinfo.ut.ee/primer3-0.4.0/primer3/input.htm), and primers (sense and antisense) were commercially synthesized at Life Technologies Corporation (Tokyo, Japan). The exonic fragments for the *hOSCP1* gene were generated using primer sequences shown in Table 1. PCR amplifications were performed in a total volume of 20 μL containing 1 μM each primers, 2.5 U Ampli*Tag* Gold DNA polymerase and 100 ng of genomic DNA. The PCR conditions were pre-denaturated at 94 °C for 7 min, followed by 35 cycles of denaturation at 94 °C for 1 min, annealing for 1 min, and extension at 72 °C for 1 min, and then final extension at 72 °C for 7 min. The annealing temperatures for the detection of hOSCP1 SNPs are summarized in Table 1. 400 ng of PCR products was used and sequenced using BigDye Terminator v3.1 Cycle Sequencing Kit (Applied Biosystems, Foster City, CA, U.S.A.) and automated Applied Biosystems 310 and 3500xl DNA sequencer. PCR was repeated and bidirectional sequencing was performed to rule out PCR-induced mutations and sequencing artifacts.

### Statistical analysis

Hardy–Weinberg equilibrium (HWE) test was performed using SNPAlyze ver. 8.0.2. (Dynacom, Chiba, Japan). Differences in allele and genotype frequencies were analyzed and compared with the data of HapMap-JPT and pilot_1_CHB + JPT_low_coverage_panel using the chi square test and Fisher's exact test. A p-value (p < 0.05) was considered to be statistically significant. All data were analyzed with SPSS (Ver. 10.0J, SPSS Inc.) software.

## Results

Table 2 summarizes the characteristics of all participants in this study. These data were collected retrospectively from their medical records after hepatectomy. Of the 18 patients, 12 were male and 6 were female; their mean age was 69.1 years. There were 9 patients with primary LC (3 patients with hepatocellular carcinoma and 6 patients with intrahepatic cholangiocarcinoma) and 9 patients with colorectal cancer liver metastasis.

Table 3 shows the results of genotype and allele frequencies of the *hOSCP1* gene. As shown in Table 3, we observed that 5 cSNPs [rs34409118 (Thr^131^ → Ala), rs61308377 (Tyr^209^ → His), rs1416840 (Ile^219^ → Thr), rs16822954 (Ser^193^ → Ser), and rs2275477 (Gly^307^ → Arg)] exhibit a genetic variation in the *hOSCP1* gene. On the other hand, no genetic polymorphisms were observed in the other 36 SNPs (Table 3). Based on these results, we next analyzed genotype and allele frequencies of these 5 cSNPs [rs34409118 (Thr^131^ → Ala), rs61308377 (Tyr^209^ → His), rs1416840 (Ile^219^ → Thr), rs16822954 (Ser^193^ → Ser), rs2275477 (Gly^307^ → Arg)] and made a comparison with a database of dbSNPs. As shown in Table 4, we found that the genotype frequency in rs34409118 (Thr^131^ → Ala) was statistically significant (p < 0.05). Likewise, significance was also observed in rs1416840 (Ile^219^ → Thr) and rs16822954 (Ser^193^ → Ser) when compared with dbSNP data. Thus, we found that there exists a significant genotype frequency in rs34409118 (Thr^131^ → Ala), rs1416840 (Ile^219^ → Thr) and rs16822954 (Ser^193^ → Ser) of the *hOSCP1* gene between the database of dbSNPs and this study. We did not find a significant difference in rs2275477 (Gly^307^ → Arg) when compared with dbSNPs (p = 0.093). Of great interest was that no heterozygous mutation in rs1416840 and rs16822954 was observed. With respect to the allele frequencies, statistical significance was seen in rs34409118 (Thr^131^ → Ala, p = 0.022) when compared with the database of dbSNPs (Table 4).

## Discussion

The present study is the first report concerning genetic polymorphism of the gene coding for human organic solute carrier protein 1, hOSCP1, in Japanese patients with non-viral liver carcinoma (LC). Although, the gene polymorphism of hOSCP1 in exon 1 (Glu^58^ → Gly) has been reported to be associated with nasopharyngeal carcinoma in Chinese patients (Nie et al., 2003), we found a significant difference of the genotype frequencies in rs34409118 (Thr^131^ → Ala), rs1416840 (Ile^219^ → Thr), and rs16822954 (Ser^193^ → Ser) in non-viral LC of Japanese patients. Specifically, we found that no heterozygous mutations in rs1416840 (Ile^219^ → Thr) and rs16822954 (Ser^193^ → Ser) are found in the present study, suggesting that among non-viral LC Japanese patients, genetic variation of the *hOSCP1* gene such as rs1416840 and rs16822954 may be an important factor in the prediction of non-viral LC occurrence.

Unexpected drug response or reduced drug efficacy is believed to be caused by genetic variations in the clinical setting. To date, investigators have reported that inherited differences in the gene expression or function of various drug transporters probably account for one of the significant reasons for this variation (Evans and Relling, 1999, Kerb, 2006, Lockhart et al., 2003). For example, Hoffmann et al. have reported that 3435C > T polymorphism in multidrug-resistance 1 (MDR1) gene resulted in the highest digoxin plasma concentration levels (Hoffmeyer et al., 2000). Likewise, a SNP of the *OATP1B1* (*OATP*-*C*[*SLCO1B1*]) gene such as T521C (Val^74^ → Ala) is likely to be associated with altered pharmacokinetics of pravastatin (Nishizato et al., 2003; Siccardi et al., 2010). Ichida et al. reported that the G774A mutation in human urate transporter 1 (hURAT1 [*SLC22A12*]) causes renal hypouricemia (Ichida et al., 2008). Thus, genetic polymorphisms of the transporter genes are likely to be associated with the occurrence of some diseases or altered pharmacokinetics responses of clinically used drugs. On the basis of these findings, therefore, we investigated the relationship between gene polymorphism of hOSCP1 and Japanese patients with non-viral LC. We found that 4 non-synonymous mutations [rs34409118 (Thr^131^ → Ala), rs61308377 (Tyr^131^ → His), rs1416840 (Ile^219^ → Thr), and rs2275477 (Gly^307^ → Arg)] and 1 synonymous mutation [rs16822954 (Ser^193^ → Ser)] are localized in the *hOSCP1* gene of non-viral LC Japanese patients. Among these, non-synonymous SNPs (rs34409118, rs61308377, rs1416840, and rs2275477) will be most likely to influence the function of the transport activity. This hypothesis that mutation in the *hOSCP1* gene has an influence on the transport activity must be confirmed; however, synonymous mutation (Ser^193^ → Ser) may have no influence on the transport activity mediated via hOSCP1. These results suggest that in order to understand the effect of SNPs in the *hOSCP1* gene on drug handling, it is important to consider the entire set of the amino acid sequence of this transport protein. It will be particularly interesting to determine if individuals with non-synonymous SNPs in the *hOSCP1* gene have altered handling of organic solutes. Such a genetic variation also suggests that there may be clinically significant differences between non-viral LC and normal human subjects with a risk of developing toxic and adverse drug reactions to several prescribed medications so that hOSCP1 mediated the transport of clinically used drugs (Kobayashi et al., 2005). Since we have revealed that hOSCP1 protein is localized in the basal membrane of the syncytiotrophoblast of human placenta (Kobayashi et al., 2005), an increase or decrease of transport activity may place a fetus at a higher risk of utero toxicity if exposed to certain exo- and endogenous compounds.

Nie et al. have reported that gene polymorphism of NOR1 in exon 1 (Glu^58^ → Gly) has been reported to be associated with nasopharyngeal carcinoma in Chinese patients (Nie et al., 2003). The genes of both hOSCP1 and NOR1 exhibit > 98% similarity at the cDNA sequence level; therefore, we subsequently analyzed whether Glu^58^ → Gly polymorphism in the *hOSCP1* gene is observed in Japanese patients with non-viral LC. However, no genetic polymorphism was found in the *hOSCP1* gene at this position (data not shown), suggesting that Glu^58^ → Gly polymorphism in the *hOSCP1* gene may not be associated with non-viral LC occurrence in Japanese patients. In this respect, a greater number of non-viral LC patients may be tested to confirm this.

Kudo et al. and Yasui et al. have revealed that reduced human organic anion transporter 2 (hOAT2[*SLC22A7*]) transporter expression in the liver indicates a significant risk for HCC development in patients with chronic hepatitis C (Kudo et al., 2013, Yasui et al., 2014). Their results suggest that reduced expression of hOAT2[*SLC22A7*] protein in non-cancerous liver tissues is significantly associated with a high incidence of HCC recurrence when compared with non-HCC patients. Although the mRNA expression of the *hOSCP1* gene was slight in normal human liver tissues (Kobayashi et al., 2005), it would be interesting to elucidate an individual mRNA expression of this gene or protein levels in non-viral LC patients.

Of the relationship between mutation of the transporter genes and the non-viral HCC, Chang et al. have revealed that mutations (IVS6 + 5G > A, c.851del4) of the aspartate glutamate carrier 2 (*AGC2*[*SLC25A13*]) gene were observed in 12% of non-viral HCC patients in Taiwan (Chang et al., 2011). In the present study, three patients out of nine primary LC subjects were HCC; further study is needed to investigate whether similar correlations between the *AGC2*[*SLC25A13*] gene mutation and non-viral HCC patients in Japanese patients are observed.

## Conclusion

We describe genetic polymorphism of the *hOSCP1* gene in Japanese patients with non-viral LC. Although there were a limited number of patients, the most notable result was to demonstrate that there exist significant differences in the genotype frequency of rs34409118, rs1416840, and rs16822954 between the database of dbSNPs and this study. Specifically, we found that there are no heterozygous mutations in rs1416840 and rs16822654 of the *hOSCP1* gene. Although several factors associated with non-viral LC development have been reported (Hamed and Ali, 2013), our results are expected to facilitate research on the prediction and susceptibility of non-viral LC in Japanese patients, and may imply whether the genetic polymorphisms of the *hOSCP1* gene are risk factors in the pathogenesis of non-viral LC or in the occurrence of non-viral LC in Japanese. Studies with a greater number of participants are necessary to clarify the relationship between the *hOSCP1* gene polymorphism and non-viral LC occurrence or recurrence in Japanese patients.

## Figures and Tables

**Table 1:** Primer sequences and PCR conditions used for genotyping of the *hOSCP1* gene.

dbSNP or oligo name	Allele change	Amino Acid Change	Forward primer (5′ to 3′)	Reverse primer (5′ to 3′)	Length (bp)	Annealing temperature (°C)
rs17851613^☨^	A → G	Leu^82^Leu	TCATGTGACCCCAGTCTATGAT	AAGAAGGCCCTGAGGACTGT	156	57
rs34409118	A → G	Thr^131^Ala	GACCTGATGACCATGGCTTT	ATGGACACGGATTGCTTTTC	313	57
rs16822954	A → G	Ser^193^Ser	TTTCCTAGCACCCCAAACAT	AATAACGGTCGCTTTGTGTTG	116	60
rs61308377	A → G	Tyr^209^His	CTTTGTCCATGGAAGGGAAA	GCCCAGCCAAAATAAAACAA	417	57
rs1416840	A → G	Ile^219^Thr	GTTTCAGGACTCGGTCTCCA	CCCAGTTCCCCAGGACTAAT	388	57
rs2359016	C → T	Lys^222^Glu	TCAGGACTCGGTCTCCATAAA	AGCCTCCAGGACTACCTAAG	510	60
rs17442970	A → C	Val^253^Leu	CAGGGGAAATGTTGGCTAGA	AACGCCAAACATAAGGCAAC	557	57
rs2275477	C → T	Gly^307^Arg	TGGAAGATTCAGGCCATTTC	ATGTGATTGCCTTCATGTGG	363	58
rs17851612	C → T	Ser^367^Gly	CACCATCCAGCAGCCTCT	TCCCTCATAGGACCAGCAAC	176	53

Positions are relative to the ATG start site and are based on the cDNA sequence from GenBank accession number AB079075.

rs17851613 has merged into rs6978099.

**Table 2:** clinical characteristics of patients with non-viral liver carcinoma.

Characteristics	
Gender (male/female)	12/6
Age (years)	69.1 (49–83)
Primary or metastatic	
Primary	9
Metastatic	9
Child–Pugh (A/B/C)	17/1/0
AST (IU/L)	30.1 (17–52)
ALT (IU/L)	24.4 (11–55)
LDH (IU/L)	287.1 (137–1284)
Cholinesterase (IU/L)	224.8 (110–285)
γ-GTP (IU/L)	90.8 (14–268)
Total protein (g/dL)	6.8 (2.0–8.2)
Albumin (g/dL)	3.8 (2.8–4.5)
Total bilirubin (mg/dL)	0.7 (0.4–0.9)
Platelet (× 10^4^/μL)	20.9 (7.6–26.6)
Prothrombin time (%)	95.5 (75–100)
ICG at 15 min (%)	17 (5–39)
Alcohol (yes/no/unknown)	9/6/3
Smoking (+/−/unknown)	6/9/3

AST, aspartate aminotransferase; ALT, alanine aminotransferase; LDH, lactate dehydrogenase; γ-GTP, γ-glutamyltranspeptidase; ICG, indocyanine green test.

**Table 3:** Genotype and allele frequencies of hOSCP1 variants.

dbSNP	Genotype			Allele	
w/w	w/m	m/m	w	m
rs77019703	C/C (1.000)	C/T (0.000)	T/T (0.000)	C (1.000)	T (0.000)
rs140615314	G/G (1.000)	G/T (0.000)	T/T (0.000)	G (1.000)	T (0.000)
rs142047873^a^	C/C (1.000)	C/T (0.000)	T/T (0.000)	C (1.000)	T (0.000)
**rs17851612**^b^	T/T (1.000)	T/C (0.000)	C/C (0.000)	T (1.000)	C (0.000)
**rs34409118**^b^	A/A (0.444)	A/G (0.167)	G/G (0.389)	A (0.528)	G (0.472)
rs146099595^b^	T/T (1.000)	T/C (0.000)	C/C (0.000)	T (1.000)	C (0.000)
rs142000188^b^	C/C (1.000)	C/G (0.000)	G/G (0.000)	C (1.000)	G (0.000)
rs139145594^b^	A/A (1.000)	A/G (0.000)	G/G (0.000)	A (1.000)	G (0.000)
rs115388124^c^	C/C (1.000)	C/T (0.000)	T/T (0.000)	C (1.000)	T (0.000)
**rs61308377**^b^	A/A (0.722)	A/G (0.167)	G/G (0.111)	A (0.806)	G (0.194)
rs138115239^b^	T/T (1.000)	T/C (0.000)	C/C (0.000)	T (1.000)	C (0.000)
**rs17442970**^b^	C/C (1.000)	C/A (0.000)	A/A (0.000)	C (1.000)	A (0.000)
**rs2359016**^b^	T/T (1.000)	T/C (0.000)	C/C (0.000)	T (1.000)	C (0.000)
**rs1416840**^b^	A/A (0.667)	A/G (0.000)	G/G (0.333)	A (0.667)	G (0.333)
**rs16822954**^a^	A/A (0.111)	A/G (0.000)	G/G (0.889)	A (0.111)	G (0.889)
rs151311655^b^	G/G (1.000)	G/T (0.000)	T/T (0.000)	G (1.000)	T (0.000)
rs151147935^b^	C/C (1.000)	C/T (0.000)	T/T (0.000)	C (1.000)	T (0.000)
rs148820896^b^	G/G (1.000)	G/A (0.000)	A/A (0.000)	G (1.000)	A (0.000)
rs148013964^b^	C/C (1.000)	C/T (0.000)	T/T (0.000)	C (1.000)	T (0.000)
rs150459319^a^	G/G (1.000)	G/A (0.000)	A/A (0.000)	G (1.000)	A (0.000)
**rs17851613**^a^	C/C (1.000)	C/A (0.000)	A/A (0.000)	C (1.000)	A (0.000)
rs144130136^a^	G/G (1.000)	G/A (0.000)	A/A (0.000)	G (1.000)	A (0.000)
rs144226900^b^	C/C (1.000)	C/T (0.000)	T/T (0.000)	C (1.000)	T (0.000)
rs14521649	G/G (1.000)	G/A (0.000)	A/A (0.000)	G (1.000)	A (0.000)
rs146088740^b^	T/T (1.000)	T/A (0.000)	A/A (0.000)	T (1.000)	A (0.000)
rs114554640	C/C (1.000)	C/T (0.000)	T/T (0.000)	C (1.000)	T (0.000)
rs150491917^a^	C/C (1.000)	C/G (0.000)	G/G (0.000)	C (1.000)	G (0.000)
rs146439686^a^	C/C (1.000)	C/G (0.000)	G/G (0.000)	C (1.000)	G (0.000)
rs149060854^b^	G/G (1.000)	G/A (0.000)	A/A (0.000)	G (1.000)	A (0.000)
rs138506112^b^	C/C (1.000)	C/T (0.000)	T/T (0.000)	C (1.000)	T (0.000)
rs148484503^a^	G/G (1.000)	G/A (0.000)	A/A (0.000)	G (1.000)	A (0.000)
rs11547025^b^	C/C (1.000)	C/A (0.000)	A/A (0.000)	C (1.000)	A (0.000)
rs141201826^b^	C/C (1.000)	C/T (0.000)	T/T (0.000)	C (1.000)	T (0.000)
rs138864592^a^	G/G (1.000)	G/A (0.000)	A/A (0.000)	G (1.000)	A (0.000)
**rs2275477**^b^	C/C (0.445)	C/T (0.444)	T/T (0.111)	C (0.667)	T (0.333)
rs140749623^b^	T/T (1.000)	T/A (0.000)	A/A (0.000)	T (1.000)	A (0.000)
rs139567315^a^	G/G (1.000)	G/A (0.000)	A/A (0.000)	G (1.000)	A (0.000)
rs182337804^b^	T/T (1.000)	T/C (0.000)	C/C (0.000)	T (1.000)	C (0.000)
rs186694883^b^	G/G (1.000)	G/C (0.000)	C/C (0.000)	G (1.000)	C (0.000)
rs150943653^b^	C/C (1.000)	C/T (0.000)	T/T (0.000)	C (1.000)	T (0.000)
rs144344454^a^	C/C (1.000)	C/T (0.000)	T/T (0.000)	C (1.000)	T (0.000)

Bold, PCR primer SNPs.

a Synonymous.

b Non-synonymous.

c Stop codon.

**Table 4:** Genotype and allele frequencies of rs34409118, rs61308377, rs1614840, rs16822954, and rs2275477 in the *hOSCP1* gene.

SNP ID	Genotype	This study	dbSNPs	p-Value^a^	Allele	This study	dbSNPs	p-Value^b^
rs34409118	A/A	0.444 (8)	0.578 (52)	< 0.01^c^	A	0.528 (19)	0.744 (67)	0.022^c^
A/G	0.167 (3)	0.333 (30)	G	0.472 (17)	0.256 (23)
G/G	0.389 (7)	0.089 (8)			
rs61308377	A/A	0.722 (13)	–	N.D.	A	0.806 (29)	0.733 (88)	0.511^d^
A/G	0.167 (3)	–	G	0.164 (7)	0.267 (32)
G/G	0.111 (2)	–			
	A/A	0.667 (12)	0.546 (94)	< 0.01^c^	A	0.667 (24)	0.756 (130)	0.298^c^
rs1416840	A/G	0.000 (0)	0.419 (72)	G	0.333 (12)	0.244 (42)
	G/G	0.333 (6)	0.035 (6)			
	A/A	0.111 (2)	0.047 (4)	< 0.01^c^	A	0.111 (4)	0.223 (19)	0.207^c^
rs16822954	A/G	0.000 (0)	0.372 (32)	G	0.889 (32)	0.767 (66)
	G/G	0.889 (16)	0.581 (50)			
rs2275477	C/C	0.445 (8)	0.547 (94)	N.S.	C	0.667 (24)	0.750 (129)	0.305^c^
C/T	0.444 (8)	0.407 (70)	T	0.333 (12)	0.250 (43)
T/T	0.111 (2)	0.046 (8)			

SNPs, single nucleotide polymorphisms. In parentheses are the numbers of subject. N.D., not determined; N.S., not significant.

a Chi-square test.

b Fisher's exact test. dbSNPs, database of SNPs (http://www.ncbi.nlm.nih.gov/snp/).

c HapMap-JPT.

d Pilot 1 CHB + JPT low coverage panel.
